# The potential of sweet potato biorefinery and development of alternative uses

**DOI:** 10.1007/s42452-021-04369-y

**Published:** 2021-02-18

**Authors:** Joana Antunez Rizzolo, Adenise Lorenci Woiciechowski, Antonio Irineudo Magalhães Júnior, Luis Alberto Zevallos Torres, Carlos Ricardo Soccol

**Affiliations:** Department of Bioprocess Engineering and Biotechnology, Federal University of Paraná (UFPR, P.O. box 19011, Curitiba, Paraná 81531-990 Brazil

**Keywords:** Sweet potato, Ethanol, Fermentation, Distilled sprit

## Abstract

The bioethanol production from the sweet potato variety BRS Cuia using three different strains of *Saccharomyces cerevisiae* (LPB1-93, ATCC-26602, and CA-11) was carried out in this research*.* Comparative analyses of consumed sugar, ethanol yield, and productivity (in tons per hectare) increased along with the concentration of cells in the inoculum. Additionally, to verify the aromatic quality of a potential sweet potato distilled spirit, volatile organic compounds were analyzed. The results showed a yield of over 90% ethanol. It was observed that the sugar consumption and ethanol production rates can be increased with a higher initial concentration of cells. This resulted in higher concentrations of ethanol in shorter times. From 100 g of the sweet potato variety BRS Cuia, the highest concentration of ethanol obtained was 25.74 g L^−1^ using the LPB1-93 strain. The estimated bioethanol production is about 10,000 L ha^−1^, with two sweet potatoes crops in a year. The ethanol production from the sweet potato variety BRS Cuia is viable, representing a sustainable alternative to fuel bioethanol, as well as an alcoholic beverage due to the volatile organic compounds present in the distilled fraction.

## Introduction

**Fig. 1:**
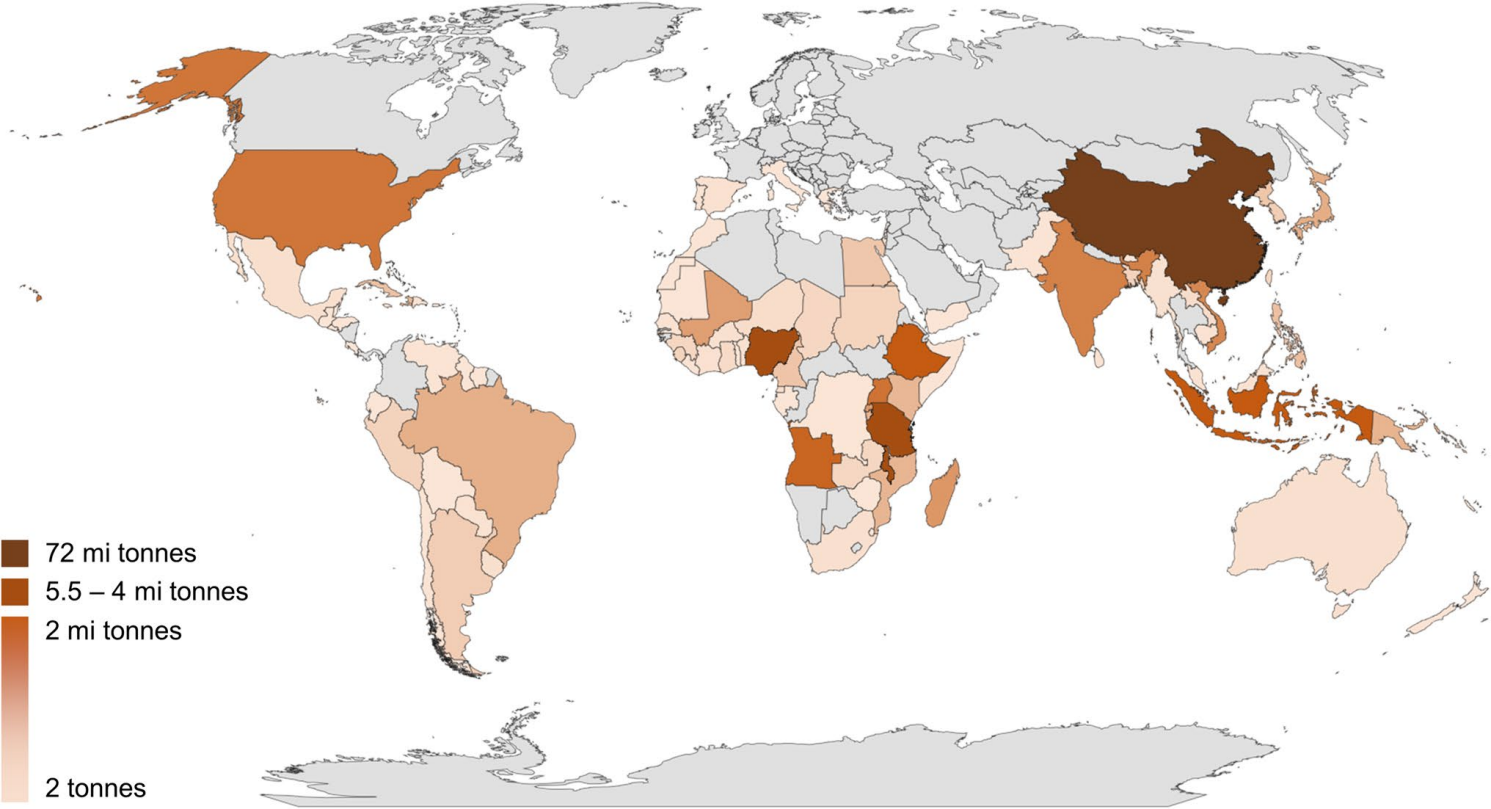
World sweet potato production in 2017 (adapted from FAOSTAT, 2017). (By Bing platform, GeoNames, HERE, MSFT, Microsoft, NavInfo, Wikipedia)

Despite the low interest in sweet potatoes, *Ipomoea potatoes* (L.), because it is a subsistence crop, it presents a high potential for ethanol production due to the production cost and good productivity [1]. The Brazilian annual production of sweet potato is about 110 thousand tons [2]. Sweet potatoes have great resistance and excellent agricultural performance, in tropical conditions [3]. However, the offer is still very centered, about 64% of global production in China, such as shown in Fig. 1 [1]. In these regions, besides being used for human food due to its good nutritional content, sweet potatoes are of great importance for the industrial production of flour, starch, and ethanol [4] and as raw material to obtain industrialized products with higher added value as dehydrated chips, cereals, jams, jellies, flour, and pasta [5]

Ethanol is a substance with many applications in the chemical and pharmaceutical industry being part of distilled and fermented beverages and, more recently, it has been widely used as a hand sanitizer in the COVID-19 pandemic crisis [6]. The bioethanol production, to be used as biofuel, is an important option environmentally friendlier than fossil fuels [7]. In addition, the raw materials used to produce bioethanol, such as sugarcane juice and corn starch, are cheaper than the fossil products [8]. Due to the depletion of energy resources, the search for alternative energy sources to meet the current demand is pivotal for environmental research. Thus, biorefineries play an important role for the generation of renewable energy source, together with products of great industrial value, which can be converted through physical, chemical, and/or biological treatments [9]. There is a growing interest in finding other alternative sources of ethanol production, such sweet potato, promoting possibilities where corn and sugarcane are not viable, bringing economic benefits to the fuel market [10].

Brazil is the second largest ethanol producer in the world, behind the USA, and one of the largest biodiesel producers. The Brazilian ethanol production was 35.6 billion liters, in 2019/2020 [11]. This represents an increase at 7.5% in relation to the previous crop. According to Brazilians Sugarcane Industry Association, the use of ethanol as biofuel, between 2003 and 2020, avoided the emission of more than 515 a million ton of CO_2_ [12]. Currently, the participation of renewable fuels in the Brazilian transport matrix is approximately 18.8% in 2018 [13].

The high agronomical yield, high starch content, and improvements in fermentation allow the sweet potatoes to be competitive with the other raw materials to produce ethanol [14]. Another competitive advantage of the sweet potato is its short life cycle, varying from 5 to 6 months, which allows two harvests per year. The global ethanol productivity depends on the fermentation yield, the concentration of fermentable sugars in the broth, and productivity of the raw materials (t ha^−1^). Brazil has been developing a large number of sweet potato varieties with high yields (t ha^−1^), with values close to the ethanol production from sugarcane and even higher in some cases. Therefore, the aim of this research was to study the potential of sweet potatoes for bioethanol production from the sweet potato variety BRS Cuia.

## Materials and methods

### Raw materials, pretreatment, and microorganism

The fresh sweet potato variety BRS Cuia was obtained from the Brazilian Agricultural Research Corporation (EMBRAPA). The moisture of the raw material was determined by drying the material in an air-circulating oven at 105 °C until constant weight. For sweet potato pretreatment, the biomass was washed and further air-dried at 60 °C in an air-circulating oven for 48 h. The hydrolysis of the material was carried out in autoclave at 121 °C for 15 min (isothermal stage) using 1.5% (v/v) dilute hydrochloric acid solution and a solid loading of 15% (w/v). The resulting hydrolysate was filtered and the pH was adjusted to 7.0 using sodium hydroxide solution at 32%.

The strains of *Saccharomyces cerevisiae* LPB1-93, ATCC-26602, and CA-11 were obtained from, respectively, Laboratory of Biotechnological Processes of Federal University of Paraná (LPB – UFPR), Agricultural Research Service (NRRL) Culture Collection, and LNF Latin-American. The culture medium used for the strains of this research was YM medium (3 g L^−1^ yeast extract, 3 g L^−1^ malt extract, 5 g L^−1^ peptone, 10 g L^−1^ glucose). Cultures were grown at 30 °C, 120 rpm and pH 7.0. For LBP1-93, ATCC-26602, and CA-11 strains, an inoculum of 10^7^ and 10^8^ cells mL^−1^ were prepared.

### Sweet potato fermentation

All *S. cerevisiae* strains were cultivated in sweet potato hydrolysate at pH 5.0, 30 °C for 24 h. The fermentation performance was assessed by consumed sugar and ethanol yield, which was estimated by the ratio of a theoretical value of ethanol yield (0.51 g g^−1^) and ethanol productivity. The solid material (yeast cells and sweet potato solids) was removed from the fermented broth by filtration.

After separation of the solid material, the liquid part (fermentation broth) was distilled, in batches. The distillation was done to recover ethanol to be used as biofuel and as distillate spirit. A traditional distillation system consisted of a heating mantle (Fisatom class 300, model 202), a 2000-mL round-bottom flask, a Liebig-type. The volatile compounds were collected at the condenser outlet. The samples were separated by temperature, the first (head) with a temperature up to 78 °C; the second (heart) with a temperature of 78.3 to 79 °C; and the third (tail) above 79 °C. Measurements of the alcoholic strength of the distilled fractions were made at 20 °C. The distilled volume was 515 mL (29° GL) of the total volume of fermented broth added to the distiller (4.4 L), resulting in 3885 L of stillage. Part of the initial total volume of 5 L was reduced in filtration (wet solids), sample removal and small losses during the entire distillation process.

### Analytical methods

Fermented samples were collected every 6 h for determination of sugars and ethanol concentrations by HPLC (Shimadzu, model LC-10AD) equipped with an Aminex HPX-87-H column and the oven operating at 60 °C. The mobile phase consisted of 5 mM H_2_SO_4_ with a flow rate of 0.6 mL min^−1^. Starch was quantified by the difference between total sugars minus the soluble sugars present in the samples.

After the distillation and recovery of the ethanol-rich fraction, volatile organic compounds (VOC) present in the distillate were analyzed in a gas chromatograph (Shimadzu, model GC17A) equipped with a HP-DB 5 column (30 m × 0.32 mm) and flame ionization detector (FID). The injector and detector temperatures were maintained at 230 °C. The initial oven temperature was maintained at 40 °C for 5 min, followed by a temperature increase to 150 °C at a rate of 20 °C min^−1^. The temperature was maintained at 150 °C for 4 min. Nitrogen was used as carrier gas at a flow rate of 1.5 mL min^−1^ and with a split ratio of 1:5.

## Results and discussion

### Ethanol production

**Fig. 2:**
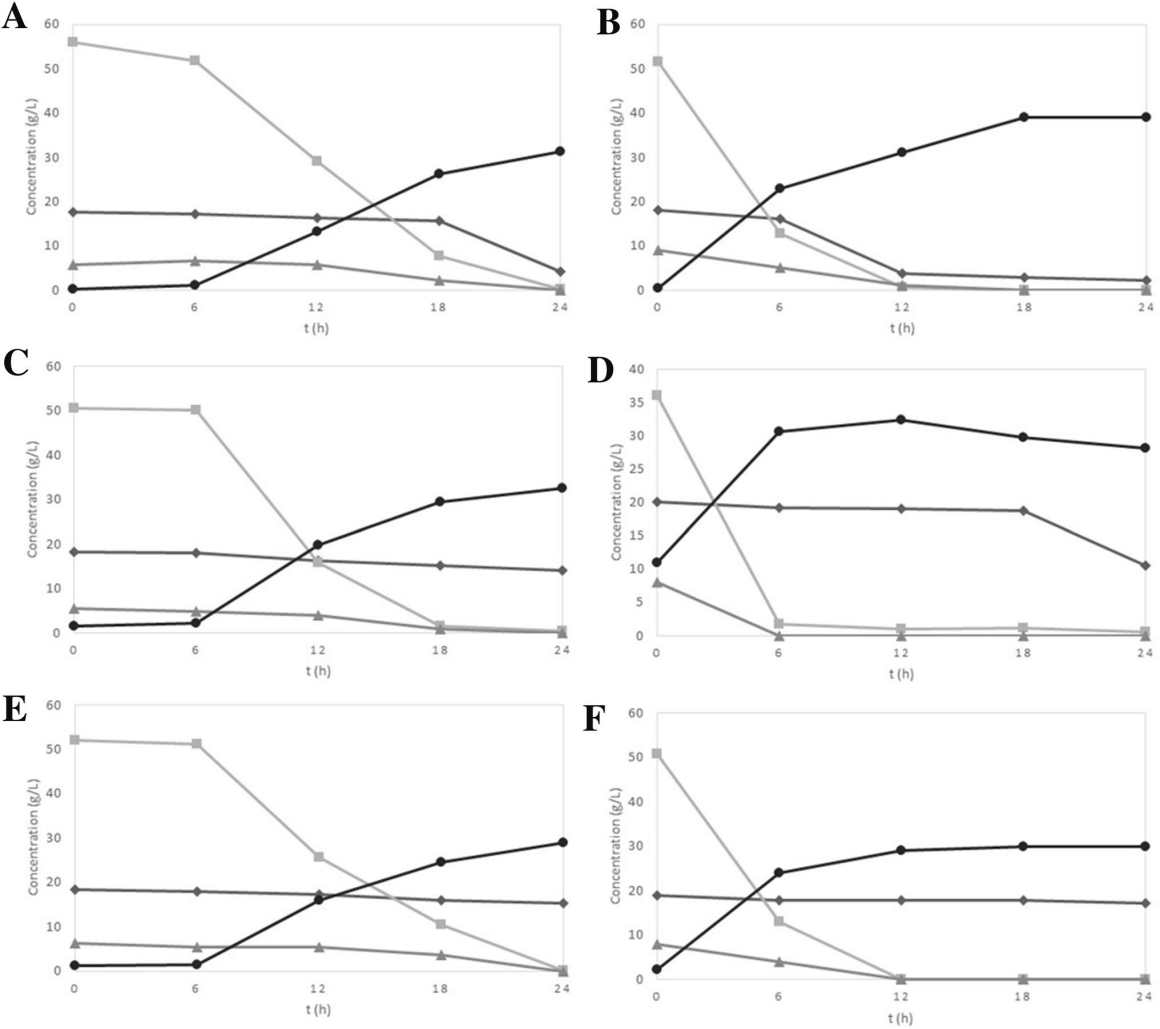
Sugar consumption from sweet potato hydrolysate and ethanol production using by different *S. cerevisiae* strains LPB1-93 (**A** and **B**), CA-11 (**C** and **D**), and ATCC-26602 (**E** and **F**) with two starting inoculum size of 10^7^ (A, C, and E) and 10^8^ (B, D, F) cells/mL at pH 5.0 and 30 °C: (square) glucose; (diamond) maltose; (triangle) fructose; and (circle) ethanol

The fermentation of sweet potato hydrolysate was carried out using three different strains of *S. cerevisiae* (LPB1-93, ATCC-26602, and CA-11) in two concentrations (10^7^ and 10^8^ cell mL^−1^) and is shown in Fig. 2. The total initial sugar concentration varied from 64.1 to 79.7 g L^−1^. After 12 h of fermentation, using an inoculum size at 10^8^ cells mL^−1^ (Fig. 2B, D, and F), glucose, the sugar in the highest concentration, and fructose were almost entirely consumed. Maltose was almost completely consumed only by the LPB1-93 strain. The ATCC-26602 strain did not consume maltose in any of the cases, giving preference to glucose and fructose. In the experiments using a lower inoculum size of 10^7^ cells mL^−1^, the sugar consumption was slower, showing that glucose was depleted between 18 and 24 h of fermentation, as well as fructose, when consumed (Fig. 2A, C, and F). It is important for the fermentation processes to be completed with minimum of sugar content or to a point where there would not be more raw material to be consumed, especially because the sugar content in the effluent can result in environmental problems, causing losses to the industry [15].

Camili and Cabelo [16], while studying the production of ethanol from cassava pulp, obtained higher ethanol concentrations after 12 to 18 h with a yeast concentration of 8% (w/w). They notice that the increase in the inoculum size has accelerated the sugar consumption and ethanol production, resulting in higher concentrations of ethanol in shorter times. The increase in cell concentration of the inoculum causes an increase in yield and productivity, by decreasing residual sugar levels [15].

The ethanol yield in this study was equal or even higher than other reports on fermentation of starch hydrolysate and other raw material hydrolysate, using yeast. Table 1 shows the fermentation performance and the main parameters analyzed, comparing the two cell concentrations applied for each strain.

**Table 1:** Fermentation parameters using *S. cerevisiae* strains with initial inoculum sizes of 10^7^ or 10^8^ cells/mL at different fermentation times

Strains	UFC (cells/mL)	10^7^				10^8^			
	Time (h)	6	12	18	24	6	12	18	24
LPB1-93	IRS^a^ (g/L)	79.7	79.7	79.7	79.7	78.6	78.6	78.6	78.6
	CS^b^ (%)	5.0	35.6	67.8	94.0	56.7	93.2	96.4	97.2
	PE^c^ (g/L)	1.2 ± 0.35	13.1 ± 0.71	25.9 ± 0.28	31.0 ± 1.27	22.6 ± 0.71	30.8 ± 0.49	38.6 ± 1.41	38.6 ± 1.41
	Y^d^ (%)	63.3	88.4	96.1	94.7	99.4	83.2	98.9	98.3
	P^e^ (L/ha/year)	5094	10,161	10,578	9,136	11,196	9,320	11,292	11,203
CA-11	IRS^a^ (g/L)	74.4	74.4	74.4	74.4	64.1	64.1	64.1	64.1
	CS^b^ (%)	1.9	51.6	76.2	80.1	67.2	68.8	68.8	82.6
	PE^c^ (g/L)	2.3 ± 0.14	18.2 ± 0.28	27.9 ± 0.07	30.9 ± 0.7	19.6 ± 0.42	21.4 ± 0.28	18.8 ± 0.07	17.2 ± 0.78
	Y^d^ (%)	92.6	77.8	90.6	94.6	89.2	95	84.1	66.8
	P^e^ (L/ha/year)	10,657	10,434	10,872	11,435	10,038	10,712	9,410	7,164
ATCC-26602	IRS^a^ (g/L)	76.8	76.8	76.8	76.8	77.8	77.8	77.8	77.8
	CS^b^ (%)	2.67	31.7	60.7	79.5	55.2	77.0	77.1	77.9
	PE^c^ (g/L)	1.6 ± 0	14.6 ± 1.06	23.3 ± 0.71	27.5 ± 0.78	21.8 ± 0.07	26.8 ± 0.35	27.8 ± 0.14	27.8 ± 0.14
	Y^d^ (%)	23.1	97.6	95.5	90.1	99.4	88	90	89.3
	P^e^ (L/ha/year)	2154	13,236	11,002	9,943	11,191	9,876	10,228	10,127

^a^Initial reducing sugar; ^b^Consumed sugar; ^c^Produced ethanol; ^d^Ethanol yield; ^e^Ethanol productivity

Li et al. [17] studied the ethanol production from sweet sorghum stem in advanced solid-state fermentation, obtaining an ethanol yield of 90.46% in continuous fermentation. From inputs of 3.72 and 16 tons of biomass, 1.54 and 6.62 tons of crude ethanol were produced, respectively. Singh et al. [18] studied the ethanol production from rice husk hydrolysate using *S. cerevisiae* with 70 g L^−1^ of initial sugar concentration and obtained 76.5% of ethanol yield and 51.4% of sugar consumption after 36 h of fermentation. Ziska et al. [19] presented an estimation of field production of ethanol from sweet potato in Maryland-USA of 8,839 L ha^−1^. Oliveira et al. [20] reported an ethanol yield of 4,379 L ha^−1^ with the sweet potato clone IPB-007; however, the other clones exhibited lower yields (1034 L ha^−1^). Masiero et al. [21] who also used Cuia sweet potato variety reported ethanol production of 4,900 L ha^−1^.

Rodrigues and Magalhães [22] investigated the production costs of a sweet potato plantation. The results showed an average productivity of 24 tons ha^−1^, while the average cost per ton harvested was approximately US$40. As for the yield of ethanol production, the average productivity was obtained from 171 L per tons and 4104 L of ethanol per hectare. Before the results to meet the required operating capacity of a small plant of 500 L per day, it will need a planting system in an area of approximately 4 hectares per month.

Jin et al. [23] studied the ethanol production from ten varieties of sweet potato, as well as the consumption of raw materials and the occupation of the soil to produce 1 ton of anhydrous ethanol. The results indicated that the two best varieties of sweet potato, called NS-007 and SS-19, presented the lower consumption of raw material (6.19 and 7.59 tons of sweet potatoes per ton of ethanol, respectively) and the lowest soil occupation (0.24 and 0.24 hectares and per ton of ethanol, respectively).

In the most optimistic scenarios, with more favorable climatic conditions, the yield of the Cuia variety reaches 60 tons ha^−1^ [24]. The present study used the average productivity value of 50 tons ha^−1^, indicating that are required 7.46 tons of sweet potato variety Cuia to produce 1 ton of ethanol and 0.15 hectares of this variety to produce 1 ton of ethanol. The raw material is responsible for half the cost of ethanol production. If less raw material were consumed, the ethanol production cost will decrease. Additionally, less land occupation not only results in lower income from the land, but also a reduced cost of cultivation. All these factors contribute to a lower cost of raw material for ethanol production [24].

Magalhães et al. [25] analyzed the sustainability of the production chain of ethanol from sweet potato in the municipality of Palmas-Brazil and made a comparison with the production of ethanol from sugarcane, from private and social perspective. The results showed that in primary production the cultivation of sweet potatoes provides greater advantages than sugarcane because the production cycle of sweet potatoes was about 5–6 months while the sugarcane was 12 months. However, it should be noted that the final gains with the sugarcane are mainly attributed to the large extensions currently being cultivated. The authors also pointed out that, in the case of sweet potato, it would be advisable to promote small producers associations for planting and cultivation to create sustainability in the production chain, lowering the production costs, which can be advantageous for small producers.

Industrial sweet potato production is not intended to be used as a food crop. They are encouraged to be cultivated to increase their starch content, significantly reducing its attractiveness as a food crop when compared to the other conventional food cultivars. Therefore, they offer potentially greater fermentable sugar yields and the opportunity to increase planted acreage (even on marginal lands) beyond what is destined for food.

### An assessment of the sweet potato bioethanol process

**Fig. 3:**
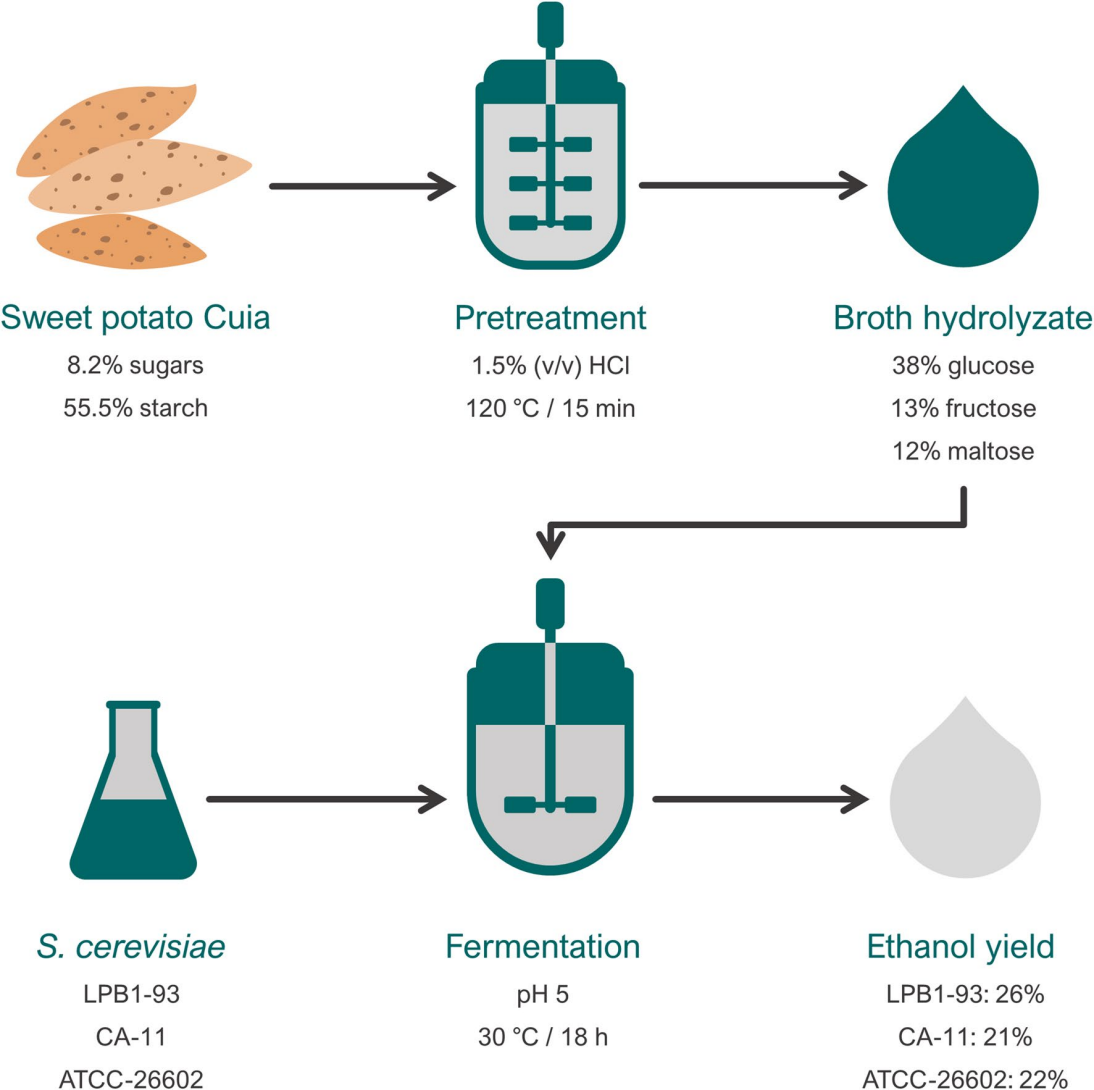
Conversion process of sweet potato into ethanol

For mass balance calculations, the input and output of the process were analyzed, measuring the quantities of fermentable sugars per 100 g of dried sweet potato variety Cuia and the quantities of ethanol produced for each strain studied. Figure 3 shows the overall mass balance of the tested strategy. Based on the data, 100 g dried sweet potato, 38 g glucose, 13.4 g fructose, and 12.3 g maltose were released in the hydrolysate after the pretreatment with dilute hydrochloric acid. After that, the hydrolysate was fermented using the yeast *S. cerevisiae* LPB1-93, CA-11 and ATCC-26602 and produced 25.74, 21.2, and 21.9 g L^−1^ of ethanol, respectively.

There is an increase in the concentration of fermentable sugars after dilute acid pretreatment, which initially were arranged as starch. The mass balance, shown in Fig. 3, indicates that the largest ethanol production (25.74 g of ethanol per 100 g of sweet potato variety Cuia) was obtained using the LPB1-93 strain. Studies conducted with sweet potatoes to produce ethanol showed yields like these investigations. Ray and Naskar [26], Swain et al. [27], and Lareo et al. [28] obtained 0.96, 1.72, and 3.87 g per 100 g of sweet potato. However, although the fermentation proposed by Lareo et al. [28] shows better results of conversion to ethanol, its estimated yield per hectare was 4790 L ha^−1^, while the Cuia variety was, due to the two harvests per year, can reach 10,000 L ha^−1^ year^−1^. The choice of different feedstocks results in different economic outcomes and risk assessment of the corresponding biomass fuel project. Thus, even if the same feedstock is used in different regions, it is unlikely to obtain the same results because most of the economic conditions such as feedstock price, plant yield, capital investment, and labor costs, vary from one region to another [29].

It is important to mention that some aspects were not addressed in this study, such as carrying out a survey of the impacts on energy consumption according to the types of equipment used, as well as the pros and cons of acid hydrolysis in relation to enzymatic processes. Therefore, it should be noted that, for future increases in scale, it is suggested to implement a survey of energy and economic consumption.

The cultivation of sweet potatoes has great aptitude for biofuel production, although in Brazil there is still a long way to go to reach a level of technology comparable to the consolidated technology of sugarcane in terms of productivity efficiency in the field. Despite this, the production of biofuel ethanol from sweet potatoes offers perspectives for the alcohol sector as an alternative to alcohol production in regions where the cultivation of sugarcane is not recommended; use of sweet potatoes as an off-season crop, in crop rotation, when the sugarcane crop is being renewed; and integration of ethanol plant-crop-agriculture, by using residues from the distillation process and the biomass of the aerial part of the plant as sources of protein for animal feed [30].

### Volatile organic compounds

The volatile organic compounds (VOCs) present in the distilled fractions recovered from the fermented sweet potato hydrolysate were analyzed by gas chromatography (GC). The alcohol content in sweet potato distillates (LPB1-93, CA-11, and ATCC 26,602) was adjusted to 36° GL. After chromatographic analyses, the following compounds were identified with their respective aromatic characteristics: ethyl acetate (fruity, sweet), 3-methyl-1-butanol (fruity banana, alcoholic), caprylic acid (waxy, desirable in some beers), ethyl decanoate (fruity apple and grape), and 1-decanol (flat, wax, sweet orange flora).

Matos [31] produced banana spirit with various yeast for the synthesis of characteristic aromas. The production of peeled banana spirit with commercial *S. cerevisiae* CA-11 showed an ethanol concentration of 414.8 mg L^−1^ (a common amount present in distilled spirits with 36° GL). However, it presented a low concentration (0.3 mg L^−1^) of isoamyl acetate (banana flavor), compared to another yeast (*Pichia kluyveri*), which produced higher amounts.

Some compounds present in the distilled spirits produced in this study (ethyl acetate, 3-methyl-1-butanol, ethyl decanoate, and 1-decanol) were also found in rum and distilled spirits from other study [32]. These commonly found aromas provide a sensory quality to the product. Comparing the VOCs (Table 2) identified in sweet potato distillates (LPB1-93, CA-11, and ATCC 26,602) with those of the banana spirit (BS) produced by Matos et al. [32], it was verified that the production of sweet potato distilled spirit is feasible, as it presents characteristic flavors also present in distilled spirits produced from other raw materials such as sugarcane. Many chemical transformations and compounds produced, such as aldehydes, acids, and esters, are related to the maturation and aging process of the distillates.

**Table 2:** Comparison of Volatile Organic Compounds of sweet potato distillates (LPB1-93, CA-11, and ATCC 26,602) with the banana spirit (BS) produced by [28] in mg/L

*S. cerevisiae* strains	LPB1-93	CA-11	ATCC 26,602	BS^a^
°GL	36	36	36	36
Ethanol	412.1	412.9	411.9	413.9
ethyl acetate	65.1	–	–	98.2
2-pentanona	–	–	–	2.8
3-methyl-1-butanol	3.7	–	–	8.2
Isoamyl acetate	–	–	–	13.5
1-decanol	–	1.3	1.9	2.7
Caprylic acid	0.8	0.4	0.7	0.3
Ethyl decanoate	0.9	0.4	0.8	0.6

^a^[28]

The oxidation and esterification of alcohols give rise to aldehydes and esters, as well as the oxidation of the lignin degradation products by ethanolysis improves the aroma and makes the flavor of the distilled spirit more pleasant. It was verified that the VOCs found in the three sweet potato distillates of this study have positive sensory characteristics for a distilled spirit. This indicates the potential to eliminate or reduce the storage time in wooden barrels with the objective of obtaining aromatic compounds as in the production of “cachaça” (sugarcane distillate drink), resulting in the reduction of one processing step and lowering the cost of the process. The presence of such VOCs indicates the possibility of producing an excellent quality distilled spirit from fermentation with sweet potato variety Cuia.

## Conclusions

Although the three studied strains of *Saccharomyces cerevisiae* (LPB1-93, CA-11, and ATCC-26602) showed to be excellent ethanol producers, the mass balance indicated that the largest biofuel production (25.74 g L^−1^ of ethanol per 100 g of the sweet potato variety Cuia) was obtained by the LPB1-93 strain. It is concluded that the ethanol production from the sweet potato variety Cuia is feasible, representing a sustainable source of income for “family farming” in Brazil, as well as a promising alternative for the development of both biofuel and distilled spirit. Therefore, the production of ethanol from sweet potato for alternative uses exhibits commercial potential and it has not yet been fully explored.

## Data Availability

Not applicable for that section.

## References

[1] Sakai P (2020). Understanding the implications of alternative bioenergy crops to support smallholder farmers in Brazil. Sustainability.

[2] FAOSTAT, Food and Agriculture Organization of United Nations, (2019). http://fao.org/faostat (accessed April 2, 2020)

[3] Kwak SS (2019). Biotechnology of the sweetpotato: ensuring global food and nutrition security in the face of climate change. Plant Cell Rep.

[4] Akoetey W, Britain MM, Morawicki RO (2017). Potential use of byproducts from cultivation and processing of sweet potatoes. Ciência Rural.

[5] Weber CT, Trierweiler LF, Casagrande T, Trierweiler JO (2018). Economic evaluation of sweet potato distilled beverage produced by alternative route. Int J Dev Sustain.

[6] Weber CT, Ranzan L, Liesegang LLM, Trierweiler LF, Trierweiler JO (2020). A circular economy model for ethanol and alcohol-based hand sanitizer from sweet potato waste in the context of COVID-19. Brazil J. Oper. Prod. Manag..

[7] Jena N, Kar MK (2020). Ethanol production from various plant sources using *Saccharomyces cerevisiae*. Int J Chem Stud.

[8] Bušić A, Mardetko N, Kundas S, Morzak G, Belskaya H, Šantek MI, Komes D, Novak S, Šantek B (2018). Bioethanol production from renewable raw materials and its separation and purification: a review, food technol. Biotechnol.

[9] Robak K, Balcerek M (2018). Review of second generation bioethanol production from residual biomass, Food Technol. Biotechnol.

[10] Schweinberger CM, Trierweiler JO, Trierweiler LF (2019). Preheating followed by simultaneous viscosity reduction, hydrolysis, and fermentation: simplifying the process of ethanol production from sweet potato, bioenergy. Research.

[11] CONAB, Monitoring the Brazilian sugarcane harvest, (2020)

[12] UNICA, Uso do etanol evita 515 milhões de toneladas de CO_2_ na atmosfera, Brazilian Sugarcane Ind. Assoc. (2010). http://e https://unica.com.br/noticias/uso-do-etanol-evita-515-milhoes-de-toneladas-de-co2-na-atmosfera/ (accessed December 17, 2020)

[13] EPE, Brazilian Energy Balance, Energy Res. Co. (2019). https://www.epe.gov.br/sites-pt/publicacoes-dados-abertos/publicacoes/PublicacoesArquivos/publicacao-377/topico-470/Relatório Síntese BEN 2019 Ano Base 2018.pdf (accessed October 18, 2020)

[14] Lareo C, Ferrari MD (2019). Sweet potato as a bioenergy crop for fuel ethanol production: perspectives and challenges. Bioethanol Prod Food Crops, Acad Press.

[15] Souza AFBC (2005) Avaliação do processo de hidrólise e fermentativo de biomassa de batata-doce [*Ipomoea batatas* (L.) Lam] por meio de células imobilizadas para produção de etanol, Federal University of Tocantins

[16] Camili EA, Cabello C (2012). Produção de etanol a partir de polpa de mandioca. Rev. Energ. Na Agric.

[17] Li S, Li G, Zhang L, Zhou Z, Han B, Hou W, Wang J, Li T (2013). A demonstration study of ethanol production from sweet sorghum stems with advanced solid state fermentation technology. Appl Energy.

[18] Singh A, Bajar S, Bishnoi NR (2014). Enzymatic hydrolysis of microwave alkali pretreated rice husk for ethanol production by Saccharomyces cerevisiae. Scheffersomyces Stipitis Their Co-Culture, Fuel.

[19] Ziska LH, Runion GB, Tomecek M, Prior SA, Torbet HA, Sicher R (2009). An evaluation of cassava, sweet potato and field corn as potential carbohydrate sources for bioethanol production in Alabama and Maryland. Biomass Bioenerg.

[20] Oliveira AM, Blank AF, Alves RP, Arrigoni-Blank MF, Maluf WR, Fernandes RP (2017). Performance of sweet potato clones for bioethanol production in different cultivation periods. Hortic Bras.

[21] Masiero SS, Peretti A, Trierweiler LF, Trierweiler JO (2014). Simultaneous cold hydrolysis and fermentation of fresh sweet potato. Biomass Bioenerg.

[22] Rodrigues LU, Magalhães KAB (2009) Levantamento dos custos para implantação do cultivo da batata-doce em reassentamentos da Enerpeixe no município de São Salvador e ou Paraná, in: 5° Semin. INICIAÇÃO CIENTÍFICA DA UFT, Tocantins

[23] Jin Y, Fang Y, Zhang G, Zhou L, Zhao H (2012). Comparison of ethanol production performance in 10 varieties of sweet potato at different growth stages. Acta Oecologica.

[24] Castro LCS, Treptow R, Becker A (2012). Potencialidade da cultivar de batata-doce BRS-Cuia como matéria-prima para a produção de etanol.

[25] Magalhães KB, Rodrigues W, da Silveira MA (2012) Análise custo-benefício social da cadeia produtiva de etanol de batata-doce no Estado de Tocantins, Universidade Federal do Tocantins

[26] Ray RC, Naskar SK (2008). Bioethanol production from sweet potato (Ipomoea batatas L.) by enzymatic liquefaction and simultaneous saccharification and fermentation (SSF) process. Dyn. Biochem. Process Biotechnol. Mol. Biol.

[27] Swain MR, Mishra J, Thatoi H (2013). Bioethanol production from sweet potato (*Ipomoea batatas* L.) flour using co-culture of *Trichoderma* sp. and *Saccharomyces cerevisiae* in solid-state fermentation. Brazil Arch. Biol. Technol..

[28] Lareo C, Ferrari MD, Guigou M, Fajardo L, Larnaudie V, Ramírez MB, Martínez-Garreiro J (2013). Evaluation of sweet potato for fuel bioethanol production: hydrolysis and fermentation. Springerplus.

[29] Yu S, Tao J (2009). Economic, energy and environmental evaluations of biomass-based fuel ethanol projects based on life cycle assessment and simulation. Appl Energy.

[30] Silva LFL, Gonçalves WM, Maluf WR, Resende LV, Lasmar R, de Carvalho C, Licursi V, Moretto P (2019). Energy and budget balances for sweet potato-based ethanol production. Pesquisa Agropecuária Brasileira..

[31] de Matos ME (2015) Produção de aguardente de banana por leveuras isoladas e selecionadas para sítese de compostos voláteis caraterísticos do aroma natural de banana, Universidade Federal do Paraná

[32] de Matos ME, Bianchi Pedroni Medeiros A, de Pereira GVM, Thomaz-Soccol V (2017). Production and characterization of a distilled alcoholic beverage obtained by fermentation of banana waste (*Musa cavendishii*) from Selected yeast. Fermentation..

